# A cross-sectional case–control study on the structural connectome in recovered hospitalized COVID-19 patients

**DOI:** 10.1038/s41598-023-42429-y

**Published:** 2023-09-21

**Authors:** Elke Lathouwers, Ahmed Radwan, Jeroen Blommaert, Lara Stas, Bruno Tassignon, Sabine D. Allard, Filip De Ridder, Elisabeth De Waele, Nicole Hoornaert, Patrick Lacor, Rembert Mertens, Maarten Naeyaert, Hubert Raeymaekers, Lucie Seyler, Anne-Marie Vanbinst, Lien Van Liedekerke, Jeroen Van Schependom, Peter Van Schuerbeek, Steven Provyn, Bart Roelands, Marie Vandekerckhove, Romain Meeusen, Stefan Sunaert, Guy Nagels, Johan De Mey, Kevin De Pauw

**Affiliations:** 1 Human Physiology and Sports Physiotherapy Research Group, Vrije Universiteit Brussel, Brussels, Belgium; 2 BruBotics, Vrije Universiteit Brussel, Brussels, Belgium; 3 Strategic Research Program ‘Exercise and the Brain in Health & Disease: The Added Value of Human-Centered Robotics’, Vrije Universiteit Brussel, Brussels, Belgium; 4 Department of Radiology and Magnetic Resonance, UZ Brussel, Brussels, Belgium; 5 Artificial Intelligence and Modelling in Clinical Science, Vrije Universiteit Brussel, Brussels, Belgium; 6 Department of Imaging and Pathology, Translational MRI, KU Leuven, Leuven, Belgium; 7 Department of Radiology, UZ Leuven, Leuven, Belgium; 8 Department of Oncology, KU Leuven, Leuven, Belgium; 9 Biostatistics and Medical Informatics Research Group, Faculty of Medicine and Pharmacy, Department of Public Health, Vrije Universiteit Brussel, Brussels, Belgium; 10 Core Facility-Support for Quantitative and Qualitative Research (SQUARE), Vrije Universiteit Brussel, Brussels, Belgium; 11 Department of Electronics and Informatics (ETRO), Vrije Universiteit Brussel, Brussels, Belgium; 12 Infectious Diseases Unit, Department of Internal Medicine, UZ Brussel, Jette, Belgium; 13 Department of Anatomical Research and Clinical Studies (ARCS), Vrije Universiteit Brussel, Brussels, Belgium; 14 Intensive Care Unit, UZ Brussel, Jette, Belgium; 15 Faculty of Psychology and Educational Sciences, Vrije Universiteit Brussel, Brussels, Belgium; 16 Faculty of Medicine and Pharmaceutical Sciences, Vrije Universiteit Brussel, Brussels, Belgium; 17 Faculty of Arts and Philosophy, University of Ghent, Ghent, Belgium

**Keywords:** Viral infection, Brain imaging

## Abstract

COVID-19 can induce neurological sequelae, negatively affecting the quality of life. Unravelling this illness's impact on structural brain connectivity, white-matter microstructure (WMM), and cognitive performance may help elucidate its implications. This cross-sectional study aimed to investigate differences in these factors between former hospitalised COVID-19 patients (COV) and healthy controls. Group differences in structural brain connectivity were explored using Welch-two sample t-tests and two-sample Mann–Whitney U tests. Multivariate linear models were constructed (one per region) to examine fixel-based group differences. Differences in cognitive performance between groups were investigated using Wilcoxon Rank Sum tests. Possible effects of bundle-specific FD measures on cognitive performance were explored using a two-group path model. No differences in whole-brain structural organisation were found. Bundle-specific metrics showed reduced fiber density (p = 0.012, Hedges’ g = 0.884) and fiber density cross-section (p = 0.007, Hedges’ g = 0.945) in the motor segment of the corpus callosum in COV compared to healthy controls. Cognitive performance on the motor praxis and digit symbol substitution tests was worse in COV than healthy controls (p < 0.001, r = 0.688; p = 0.013, r = 422, respectively). Associations between the cognitive performance and bundle-specific FD measures differed significantly between groups. WMM and cognitive performance differences were observed between COV and healthy controls.

## Introduction

During the last 3 years of the COVID-19 pandemic, over 662 million people have been infected with severe acute respiratory syndrome coronavirus 2 (SARS-CoV-2)^1,2^. Depending on the severity of the infection, the disease can cause persisting symptoms that continue to be present beyond the initial ailment, such as respiratory distress, fatigue, cognitive dysfunctions, and neurological sequelae^3–9^. The origin of the central nervous system involvement related to COVID-19 can be attributed to the virus operating via perineural invasion in olfactory mucosa^10^. The virus' spike protein binds to angiotensin-converting enzyme 2 (ACE-2) receptors on the nasal respiratory and olfactory cells^10^. It then spreads into the olfactory and sensory neurons within the central nervous system^11^. The ACE-2 receptor is present on vascular endothelial cells and smooth muscle cells in all body organs. SARS-CoV-2 binds to the ACE-2 receptor of the vascular endothelium, suppresses angiotensin (1–7) of the vascular smooth muscle, and causes oxidative stress after crossing the blood–brain barrier^11^. In turn, this reaction might lead to hypertension and ischemic cerebrovascular lesion overexpression, which reduces the functionality of the central nervous system by causing neuropathological changes and which may negatively impact the patient’s quality of life^12,13^.

Roughly one-fifth of the people with COVID-19 are estimated to develop specific (e.g. headache and fatigue) or non-specific neurological sequelae (e.g. cerebrovascular disorders and other CNS disorders)^14^. Longitudinal diffusion-weighted magnetic resonance imaging (dMRI) studies have identified brain alterations following COVID-19, including changes in local white matter microstructure and structural whole-brain reorganisation^4,15^. Using diffusion tensor imaging (DTI), decreased mean, axial, and radial diffusivity, combined with increased fractional anisotropy (FA) in the corona radiata external capsule and superior frontal-occipital fasciculus has been found in former COVID-19 patients at the 3-month rehabilitation time-point compared to healthy controls^16^. Furthermore, a DTI tractography analysis showed widespread reduction in fibre bundle volume, length, and mean FA in the association, commissural, projection, and limbic fibre bundles at three months of recovery ^17^. Using dMRI-based graph theory, Tassignon et al. (2023) identified changes in structural organisation among hospitalised COVID-19 patients, with the characteristic path length decreasing over time following the three months after hospital discharge^18^. However, no comparison with healthy controls was made^18^. Further research is therefore needed to investigate whether similar changes in the structural whole-brain organisation can also be detected cross-sectionally compared to healthy controls and whether these alterations align with previous research.

Changes in FA and global mean diffusivity of white matter and changes in grey matter volume, reported in both the acute phase of infection and in recovered patients have been correlated with cognitive disorders^12,15,16^. Cognitive dysfunctions entail problems with the executive function, including reasoning, problem-solving, spatial planning and target detection^12,19^. The cognitive problems are worrisome, as impaired cognitive functioning can lead to poor work-related and functional outcomes, and an increased risk of cognitive deterioration at a later stage of life^19–22^. However, there appear to be signs that impaired cognitive function and brain structure may partially recover over time^19,23,24^. Nevertheless, impaired cognitive functioning resulting from a SARS-CoV-2 infection may affect the perception–action coupling and alter the motor response or motor behaviour^12,25^. Alterations to the motor connectome could possibly unravel this diseases’ underlying mechanism and explain the cognitive dysfunctions. To the best of our knowledge, no studies on the motor connectome and its impact on cognitive functioning are available. Therefore, this study focuses on probing the white matter fibre microstructure and, more specifically, the fibers related to motor functioning (i.e. motor-related tracts).

To date, white matter brain microstructural alterations following a SARS-CoV-2 infection have been investigated using DTI. However, seeing that DTI assumes a single dominant fiber direction per voxel, its application disregards the presence of multiple fiber directions, estimated to be present in up to 90% of voxels at practical imaging resolutions^26^. This leads to problems with interpretation, limited biological specificity of associated metrics, and detrimental effects on processing techniques such as tractography ^27–29^. The fixel-based statistical analysis paradigm was created to solve these issues^27,30,31^. Constrained spherical deconvolution (CSD) was developed to allow for multiple fiber directions, based on which the fixel-based statistical framework was made to allow for a replacement of the voxel-based statistics^27,32^. A "fixel" is an individual fiber population within a voxel that allows fibre-specific measures to assess white matter characteristics and changes in this setting^27,30,31^. These measures include fiber density (FD), fiber cross-section (FC) and fiber density and cross-section (FDC)^27^. FD refers to the concentration of nerve fibers (i.e. axons) per fixel^27^. FC refers to the shape and size of a nerve fiber, which provides information about its gross/macroscopic structure. FDC is a modulated measure that constitutes the product of the FD and FC, providing insights into the underlying microscopic and macroscopic structure of white matter fibers^27^. To the best of our knowledge only one study using fixel-based analysis has been published so far to explore changes in the white matter microstructure following a COVID-19 infection^33^. This study found a reduction of fibre density in recovered COVID-19 patients compared to healthy controls in the posterior genu and rostral body of the corpus callosum, the arcuate fasciculus, the cingulum, the fornix, the inferior frontal-occipital fasciculus, the inferior and superior longitudinal fasciculus, the uncinate fasciculus, the corona radiata, and corticospinal tracts.

Unravelling the neural basis and impact of COVID-19 on the whole-brain structural organisation, white matter microstructure, and cognitive performance may further elucidate this illness's neurocognitive implications. Therefore, the aim of this cross-sectional controlled trial is fourfold. The first aim is to investigate the differences in whole-brain structural connectivity between COVID-19 patients and healthy volunteers who were assumed to have never had a COVID-19 infection, based on graph-theoretical measures derived from diffusion MRI. We hypothesise that structural brain connectivity will be unfavourable in COVID-19 patients compared to healthy individuals. The second aim is to investigate white matter microstructure differences related to motor functioning between COVID-19 patients and healthy individuals using a fixel-based analysis (FBA). Based on findings from DTI studies^4,12,15,16,18^, we expect a reduced FD and potentially reduced FC and FDC in the COVID patients compared to healthy volunteers. The third aim is to investigate the difference between COVID-19 patients and healthy individuals regarding cognitive performance. We hypothesise that cognitive performance will be worse in COVID-19 patients than in healthy individuals. The fourth and final aim is to investigate whether bundle specific FD measures explain differences in cognitive performance for COVID-19 patients and healthy individuals.

## Methods

### Study design, study approval and registrations

A cross-sectional single-centred case–control study was performed at the University Hospital of Brussels (UZ Brussel, Jette, Belgium) between January and March 2021. The study was executed in compliance with the Declaration of Helsinki^34^, was approved by the Medical Ethics Committee of the University Hospital of the Vrije Universiteit Brussel (B.U.N. 1432020000338) and was registered on ClinicalTrials.gov Protocol Registration and Result System (NCT04726176).

### Participants’ recruitment and consent

Twenty patients with clinical signs of COVID-19 pneumonia were included in this study. Patients were eligible to participate if they had a positive reverse transcriptase-polymerase chain reaction test (RT-PCR) and were hospitalised at the UZ Brussel. A radiology resident performed patient recruitment in collaboration with the department of infectious diseases. The Intensive Care Unit and Infectiology Department created a list of all hospitalised COVID-19 patients at the UZ Brussel to facilitate the recruitment. An infectious disease specialist contacted the patients on the list by phone or asked about the patient’s willingness to participate during a follow-up consultation. Patients who expressed their willingness to participate were contacted by the radiology resident to further explain the study protocol. In addition to the cohort of patients with COVID-19, a control group of 18 individuals (i.e. healthy controls) was included and was age- matched to the patient cohort. Individuals who never had a symptomatic COVID-19 infection were eligible for participation. The recruitment of these individuals was conducted by means of convenience sampling and through the network of the co-authors. All participants provided their written informed consent after being informed both verbally and in writing regarding the study protocol.

### Protocol

All tests and measurements were conducted at the department of Radiology-Magnetic Resonance (UZ Brussel). Participants underwent a Magnetic Resonance Imaging (MRI) brain scan and cognitive test battery. The patients with COVID-19 underwent the MRI and cognitive test battery upon hospital discharge. The MRI brain scan was acquired in a supine position using a 3 T MRI Ingenia scanner with a 32-channel head coil (Philips Medical Systems, Best, The Netherlands). The protocol for both cohorts consisted, among others, of an 3D-T1 weighted spin-echo images, dMRI (48 directions at a b-value of 3000s/mm^2^). Characteristics of the different brain imaging techniques applied are available in the paper by Tassignon et al. (2023)^18^.

### Outcome measures

#### Structural brain connectivity

Image pre-processing, structural connectome construction were executed as described by Tassignon et al. (2023)^18^. In short, an MRTRix3 pipeline, implemented in the KU Leuven neuroimaging suite (KUL_NIS, https://github.com/treanus/KUL_NIS), was used to derive SIFT2-reweighted, anatomically constrained whole-brain probabilistic tractograms with single-shell single-tissue CSD^35–37^. Grey matter parcellation was performed with Freesurfer 6.0.0. using the Desikan-Killiany atlas^38^. Next, as detailed by Tassignon et al. (2023), structural connectomes were generated from the SIFT2-reweighted tractograms and the parcellated T1 images^18^. Measures of local efficiency, global efficiency, characteristic path length and cluster coefficiency were calculated and normalized against the mean of 100 equivalent random networks.

#### White matter microstructure—whole-brain fixel based analysis

Single-shell two tissue FBA followed the steps described by Raffelt et al. 2017^31^, and detailed in the MRtrix3 user guide^39^. Briefly, fiber orientation distribution (FOD) maps were calculated for each subject based on group averaged response functions for anisotropic tissue (white matter) and isotropic tissue (grey matter and cerebrospinal fluid). Subject FOD maps were then normalized over the two tissue types and used to generate population averaged (study-specific template) FOD images. Fixels were generated from the template FOD maps and for each individual subject’s FOD maps after warping to template space. Individual subject fixels were assigned to the template fixels, then the CSD-derived metrics FD, FC, and FDC were calculated for each subject. A whole brain tractogram with 20 million streamlines was generated from the template FOD maps and filtered with SIFT to 2 million streamlines. This tractogram was used to define a fixel-to-fixel connectivity matrix, and to define a fixel mask with at least 150 streamlines per fixel. All fixel maps were smoothed based on a sparse fixel-to-fixel connectivity matrix. Finally, statistical analysis compared FD, FC (log scaled), and FDC measures between COVID-19 patients and healthy controls with fixelcfestats^30^. Fixelcfestats controls for familywise error rate using permutation testing to control for multiple testing^30^. Total intracranial volumes calculated by FreeSurfer were used as regressors of noninterest while comparing FC and FDC measures between the two groups.

#### White matter microstructure—bundle-specific CSD-derived metrics

Bundle specific tractography was carried out using the KUL_FWT pipeline^40^ in native subject space, and resulting tractograms were warped to the fixel template space, to sample the individual subject mean FD, FC, and FDC within the traversed fixels.

#### Cognitive performance

The computerized cognitive test battery "Cognition" (Joggle® Research, Seattle, WA, USA) was conducted using an iPad. The test battery has an average duration of approximately 18 min, is sensitive to multiple domains at high-level cognitive performance and has been proven to engage specific brain regions, evidenced by functional neuroimaging^41^. The test battery is compiled out of 7 tests, including the motor praxis test, visual object learning test, abstract matching, line orientation test, digit symbol substitution test, balloon analogue risk test, NBACK and psychomotor vigilance test. These tests measure sensorimotor speed, spatial learning and memory, abstraction, spatial orientation, complex scanning and visual tracking, risk decision-making, working memory, and vigilant attention. Participants practised each cognitive test once to mitigate learning effects. For this study the primary outcome of interest was the median reaction time on the motor praxis, digit symbol substitution and psychomotor vigilance tests.

### Statistical analysis

All statistical analyses were performed using R (version 4.1.2; R Core Team, 2022)^42^. A p-value below 0.05 was considered statistically significant. To investigate the first aim, the difference between COVID-19 patients and healthy controls in structural brain connectivity was assessed by means of the Welch-two sample t-test or, when the assumptions were not met, by the non-parametric Wilcoxon rank sum exact test. In particular, group differences in terms of the clustering coefficient and global and local efficiency were inspected parametrically. The characteristic path length was examined non-parametrically because of non-normality (healthy controls: Shapiro–Wilk W = 0.864, p = 0.018; COVID-19 patients: Shapiro–Wilk W = 0.741, p < 0.001). No corrections for multiple testing were applied.

For the second aim, whole-brain FBA was conducted to examine differences between COVID-19 patients and healthy controls by means of whole-brain FD, FC and FDC (Fig. 2). Subsequently, tract-specific FBA was performed to test the differences in motor-related tracts between groups terms of mean FD, FC and FDC, respectively. Hence, three multivariate analysis of variance (MANOVA) models were constructed per bundle to avoid multiple testing, using the lme4^43^ and lmerTest^44^ packages in R. All assumptions were checked and fulfilled. First, multivariate results were examined, after which univariate parameter estimates were inspected. Hedge’s g were calculated as it is a standardized mean difference measure that adjusts for potential bias in the estimation of the effect size due to small sample sizes. For the third aim, the assumptions for performing a MANOVA were checked but not fulfilled (i.e., violation of both normality and homoscedasticy). Therefore, Wilcoxon rank sum exact tests were used to analyse group differences in cognitive performance on the motor praxis, digit symbol substitution and psychomotor vigilance tests. Wilcoxon test effect sizes (r values) were calculated and are provided in the results section. No corrections for multiple testing were applied.

For the fourth aim, two-group structural equation modelling was used to unravel the associations between cognitive performance and white matter microstructure and whether these associations differ between the healthy controls and the COVID patients. Specifically, a two-group path model, grouping on participant type (healthy controls, COVID-19 patients), was constructed using the lavaan package (version 0.06-11) in R^45^. Multicollinearity between the different variables was evaluated. The correlations between the left whole pyramidal tract and left corticospinal tract (r = 0.94) and between the corresponding right measures (r = 0.94), were almost perfect, signifying redundant variables. Consequently, the left and right corticospinal tracts were dropped from the model. The robust maximum likelihood estimator was used. A stepwise model building approach was adopted. In the first model, all hypothesized paths were modelled, and paths with p-values > 0.10 in both groups were removed, resulting in a good fitting model. We manually defined parameters to test whether the parameter estimates differ significantly between both groups using Wald tests. Before interpreting the parameter estimates, we evaluated how well the proposed model fits the data using the following cut-offs. First, if the Chi-square test is non-significant, the model fit is considered acceptable as the observed covariance matric is deemed similar to the model implied covariance matric. It is advised that the Comparative Fit Index exceeds 0.90 or, preferably, 0.95^46^. For the Tucker Lewis Index, a value between 0.90 and 0.95 is considered as a marginal fit, and values exceeding 0.95 represent a good fit^47^. Concerning the Root Mean-Square Error of Approximation a value below 0.04 describes a good fit and below 0.08 a moderate fit^48^. Values of the Standardized Root Mean Square Residual exceeding 0.10 are indicative of a poor fit^48^. Statistical analyses and graphical representations were made using several R-packages^45,48–54^.

## Results

### Participants

Table 1 provides the participants characteristics. A total of 20 COVID-19 patients and 18 healthy controls were included in this study. Within the group of COVID-19 patients, one male patient dropped out due to personal reasons and one male participant did not undergo the MRI brain scan due to claustrophobia. Within the group of healthy controls, one female participant refused to undergo an MRI brain scan due to claustrophobia. Accordingly, the data of 18 COVID-19 patients and 17 healthy controls were analysed. There were no baseline age-differences between groups (p-value = 0.146).

**Table 1:** Patient characteristics.

	Covid patients	Healthy controls
Mean age ± SD (years)	56 ± 9	52 ± 11
Age range (years)	36—76	30—71
Sex (Male/Female)	n = 14 / n = 4	n = 12 / n = 5
BMI (kg/m^2^)	28.0 ± 3.8	25.6 ± 2.5
Smoking (%, n)	11%, n = 2	18%, n = 3
Mean length of hospital stay ± SD (days)	14 ± 10	NA
Neurological symptoms at the time of hospital admission		
Headache	44.4%, n = 8	NA
Concentration problems	38.9%, n = 7	NA
Fatigue	27.8%, n = 5	NA
Agitation	22.2%, n = 4	NA
Memory disorders	22.2%, n = 4	NA
Delirium	16.7%, n = 3	NA
Decreased consciousness	11.1%, n = 2	NA
Vertigo	5.6%, n = 1	NA
Neurological symptoms at the time of the experimental session		
Concentration problems	33.3%, n = 6	NA
Fatigue	16.7%, n = 3	NA
Memory disorders	22.2%, n = 4	NA
Comorbidities		
Type 2 diabetes mellitus	39%, n = 7	0%, n = 0
Overweight and obesity	78%, n = 14	77%, n = 13
Arterial hypertension	28%, n = 5	12%, n = 2
Dyslipidaemia	22%, n = 4	0%, n = 0
Migraine	6%, n = 1	0%, n = 0
Alcohol dependence	6%, n = 1	0%, n = 0
Chronic obstructive pulmonary disease (COPD)	11%, n = 2	0%, n = 0
Asthma	6%, n = 1	0%, n = 0
Fibromyalgia	6%, n = 1	0%, n = 0
Ulcerative colitis	6%, n = 1	0%, n = 0
Hypothyroidism	6%, n = 1	0%, n = 0

*NA* not applicable at the time of the experimental session.

### Aim 1: structural brain connectivity

**Figure 1:**
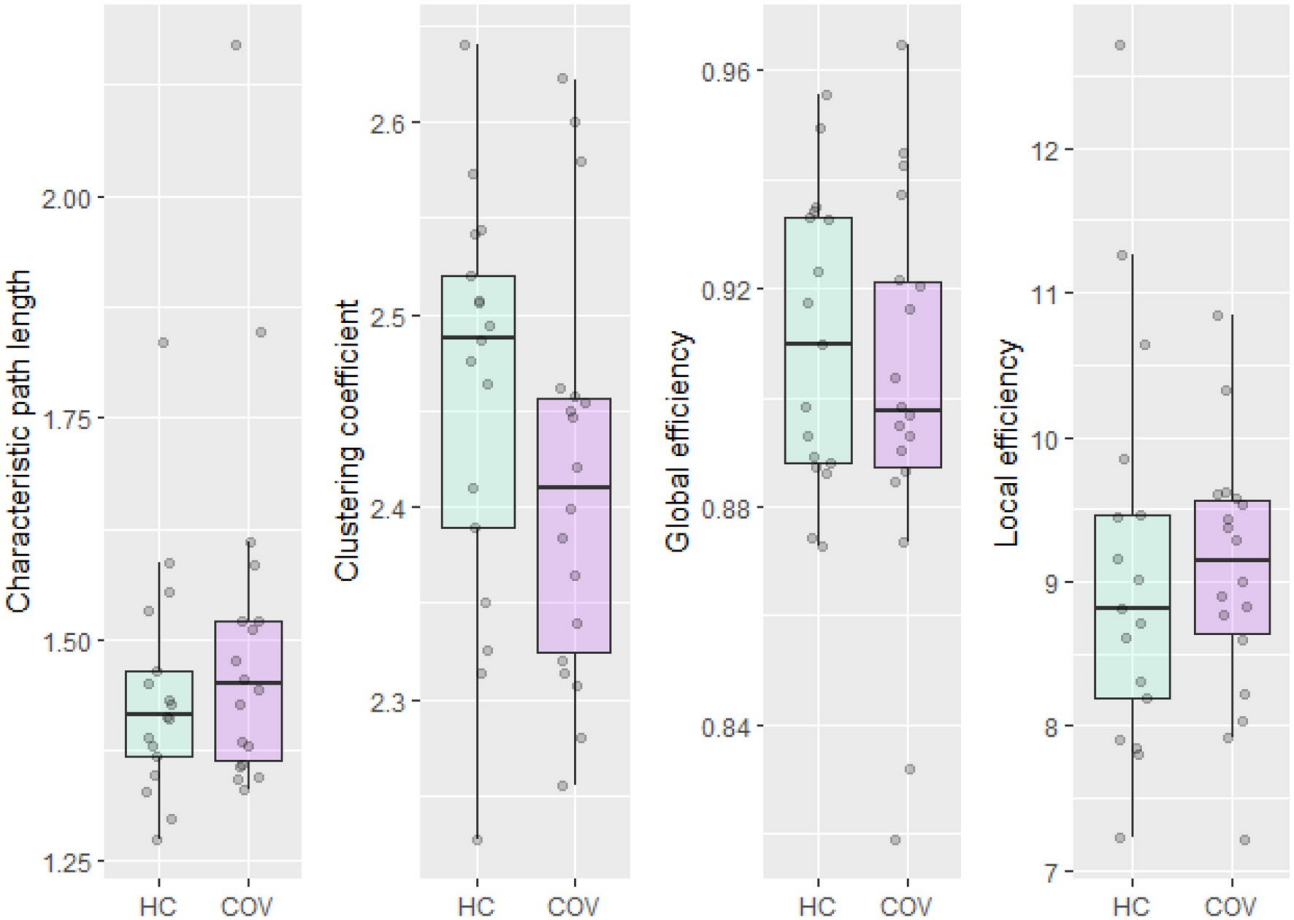
Boxplots for the graph theory measures of COVID-19 patients (COV) and healthy controls (HC). Grey dots represent individual observations.

Figure 1 shows boxplots for the normalized whole-brain structural graph metrics of the COVID-19 patients and healthy controls for each graph theory measure separately. No significant differences between COVID-19 patients and healthy controls are detected (all p > 0.24), as can be seen in Table 2.

**Table 2:** Statistical analysis of differences in graph theory measures between COVID-19 patients (n = 18) and healthy controls (n = 17).

Graph theory measure	Group	Mean ± SD	t-value^+^	p-value	95% confidence interval
Characteristic path length*	HC	NA	127^++^	0.404	−0.11 to 0.042
	COV	NA			
Cluster coefficient	HC	2.46 ± 0.11	1.186	0.244	−0.03 to 0.12
	COV	2.41 ± 0.11			
Global efficiency	HC	0.91 ± 0.03	0.876	0.388	−0.01 to 0.03
	COV	0.90 ± 0.04			
Local efficiency	HC	9.11 ± 1.39	0.136	0.893	−0.76 to 0.87
	COV	9.06 ± 0.88			

*HC* healthy controls, *COV* COVID-19 patients, *SD* standard deviation, *NA* not applicable due to non-normality.

*Wilcoxon rank sum exact test used due to outliers and non-normality.

^+^Unless stated otherwise.

^++^Wilcoxon W-value.

### Aim 2: white matter microstructure

#### Whole-brain FBA

**Figure 2:**
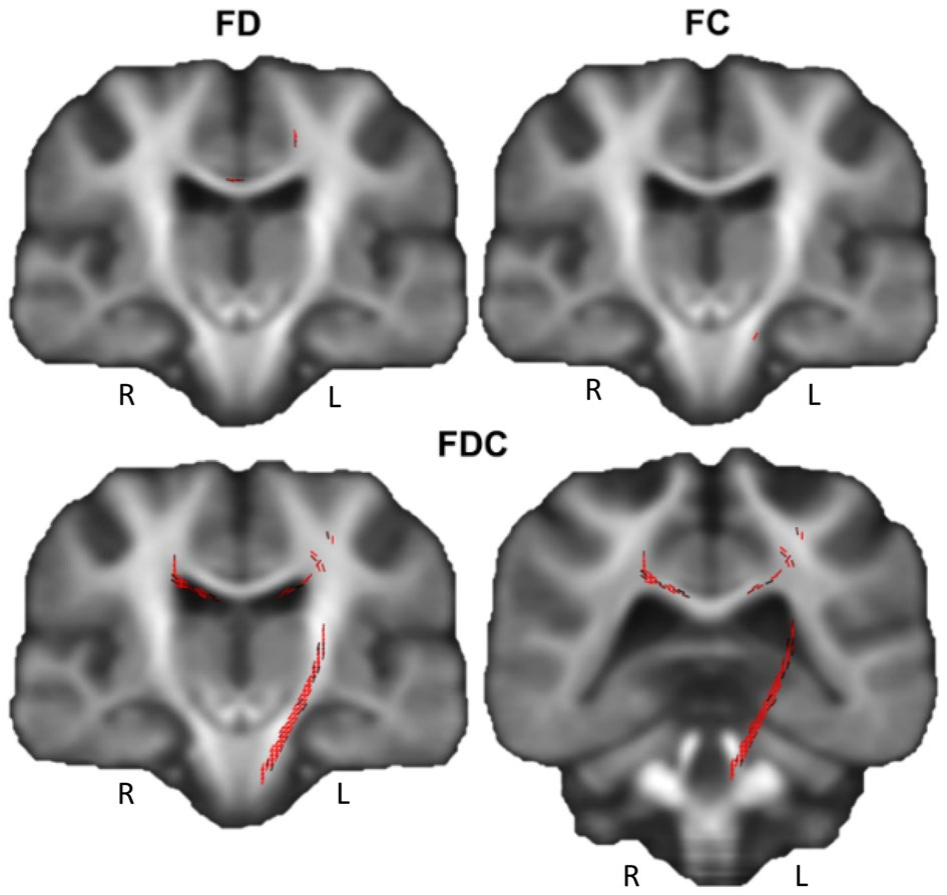
Whole-brain fixel-based analysis results for the Healthy controls > COVID-19 patients contrast showed fixel differences at p_(Free Water Elimination)_ < 0.1 significance threshold. *R* right, *L* left.

Figure 2 visualises the differences in whole-brain FD, FC and FDC between healthy controls and COVID-19 patients. A threshold of α = 0.10 was used as results with a significance value of 0.10 > p_(Free Water Elimination)_ < 0.05 were considered borderline significant. FD showed minor differences in the corpus callosum and subcortical white matter of the left precentral gyrus (medially), FC showed minimal fixel differences in the left cerebral peduncle, and FDC showed more prominent differences in the left pyramidal tract, corpus callosum (parietal segment) and subcortical white matter (precentral gyrus).

#### Bundle-specific CSD-derived metrics

**Figure 3:**
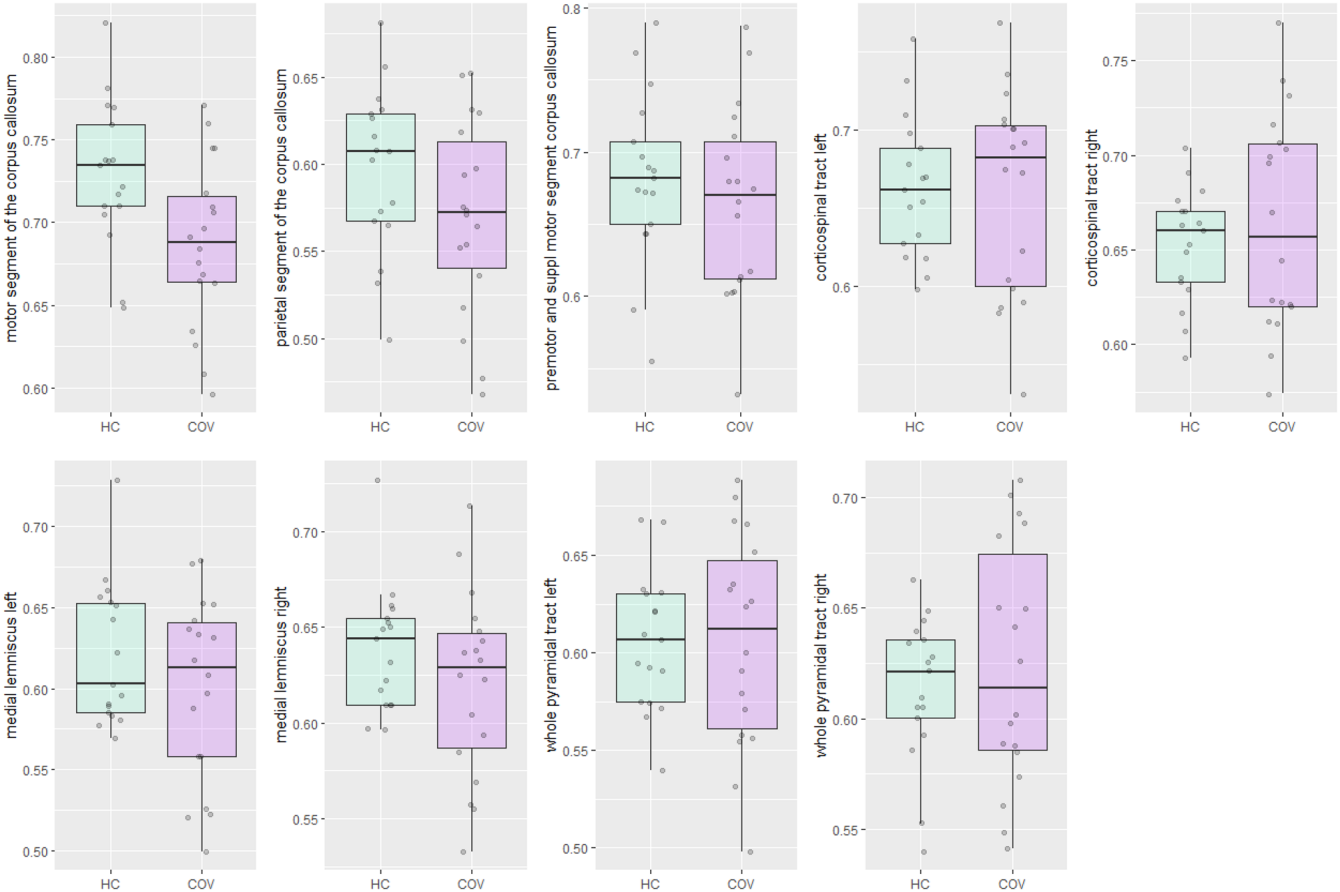
Boxplots of fiber density of the motor segment of the corpus callosum, parietal segment of the corpus callosum, premotor and supplementary motor segment of the corpus callosum, bilateral medial lemnisci, bilateral whole pyramidal tracts and corticospinal tracts in COVID-19 patients (COV) and healthy controls (HC).

Figure 3 visualises FD of the motor segment of the corpus callosum, parietal segment of the corpus callosum, premotor and supplementary motor segment of the corpus callosum, bilateral medial lemnisci, bilateral whole pyramidal tracts and corticospinal tracts in COVID-19 patients and healthy controls. A significant difference between COVID-19 patients and healthy controls was detected for the motor segment of the corpus callosum (Table 3).

**Table 3:** Results of analysis of variance models per brain region of interest of the differences in fiber density between COVID-19 patients (n = 18) and healthy controls (n = 17).

Brain region	Estimate (SE)		Adjusted R-squared	Significance level	Effect size [95% CI] (Hedges’ s g)
Corpus callosum					
Motor segment	Intercept	0.730 (0.012)	0.153	< 0.001	0.884 [0.180–1.589]
	Group	−0.043 (0.016)		**0.012**	
Parietal segment	Intercept	0.597 (0.013)	0.038	< 0.001	0.507 [−0.178 to 1.188]
	Group	−0.027 (0.018)		0.136	
Premotor area & SMA	Intercept	0.682 (0.015)	−0.009	< 0.001	0.276 [−0.401 to 0.950]
	Group	−0.018 (0.021)		0.412	
Corticospinal tract					
Left corticospinal tract	Intercept	0.663 (0.014)	−0.030	< 0.001	0.049 [−0.624 to 0.721]
	Group	−0.003 (0.019)		0.883	
Right corticospinal tract	Intercept	0.653 (0.011)	−0.014	< 0.001	−0.242 [−0.916 to 0.433]
	Group	0.011 (0.016)		0.470	
Medial lemniscus					
Left medial lemniscus	Intercept	0.621 (0.012)	0.014	< 0.001	0.401 [−0.278 to 1.080]
	Group	−0.021 (0.017)		0.234	
Right medial lemniscus	Intercept	0.639 (0.010)	0.021	< 0.001	0.433 [−0.247 to 1.113]
	Group	−0.018 (0.014)		0.199	
Whole pyramidal tract					
Left pyramidal tract	Intercept	0.605 (0.011)	−0.030	< 0.001	−0.014 [−0.687 to 0.658]
	Group	0.001 (0.016)		0.965	
Right pyramidal tract	Intercept	0.614 (0.011)	−0.017	< 0.001	−0.213 [−0.887 to 0.461]
	Group	0.010 (0.015)		0.524	

Group = COVID-19 patients, *SMA* supplementary motor area, *SE* standard error, *CI* confidence interval.

Significant values are in bold.

The multivariate ANOVA model showed a significant association between group and the corpus callosum (F(3,31) = 0.771, p = 0.042). Univariate inspection revealed only a significant association between group membership and the motor segment of the corpus callosum with a large effect size (${\widehat{\beta }}_{group}$ = −0.043, p = 0.012, Hedges’ s g = 0.884). No other statistically significant differences were found between COVID-19 patients and healthy controls for the other regions.

**Figure 4:**
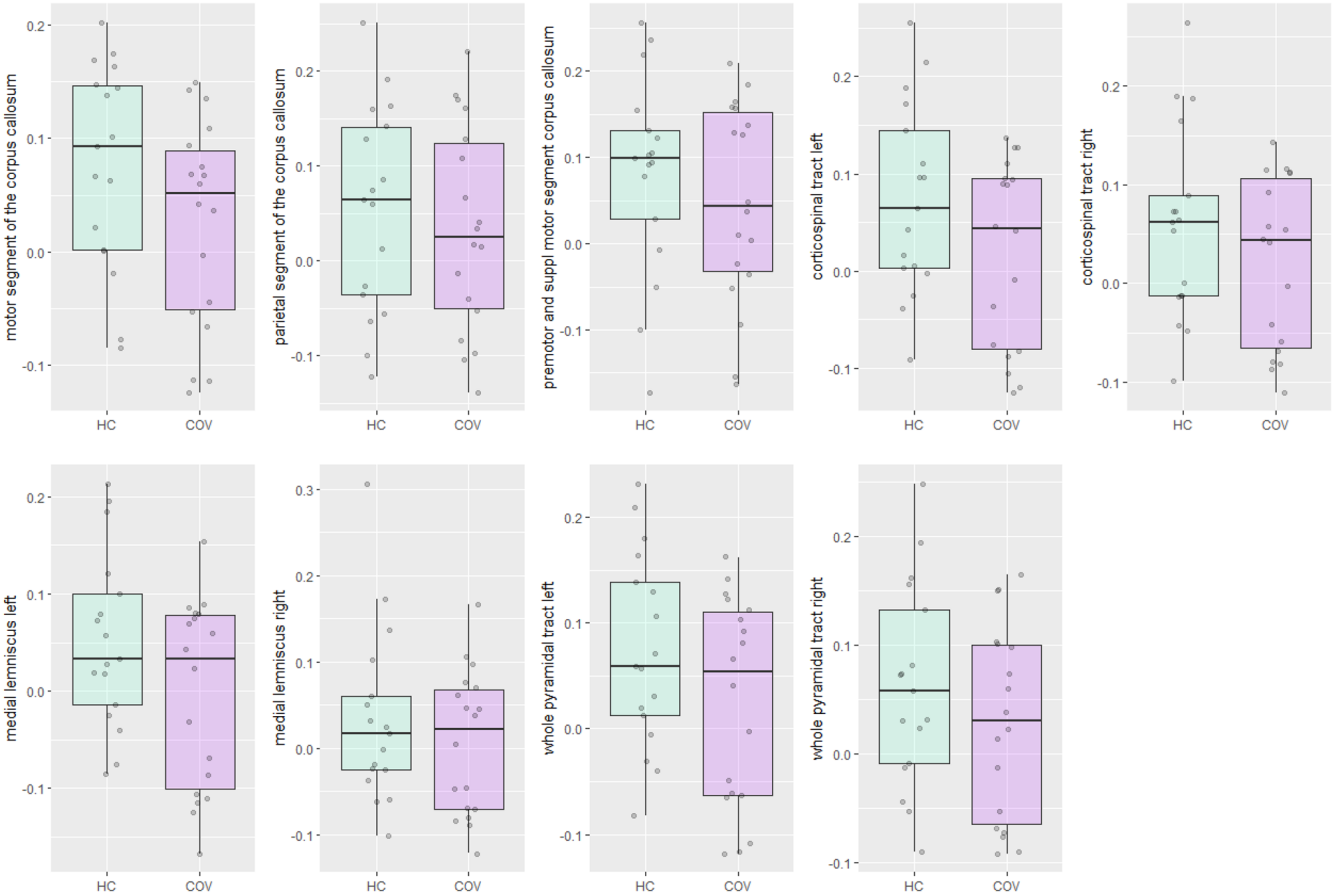
Boxplots for fiber density of the motor segment of the corpus callosum, parietal segment of the corpus callosum, premotor and supplementary motor segment of the corpus callosum, bilateral medial lemnisci, bilateral whole pyramidal tracts and corticospinal tracts in COVID-19 patients (COV) and healthy controls (HC).

Figure 4 shows the boxplots of FC of the motor segment of the corpus callosum, parietal segment of the corpus callosum, premotor and supplementary motor segment of the corpus callosum, bilateral medial lemnisci, bilateral whole pyramidal tracts and corticospinal tracts in COVID-19 patients and healthy controls. No clear differences were visually observed between both groups.

Yet, the statistical modelling showed a marginal significant effect of FC in the left corticospinal tract in COVID-19 patients compared to healthy controls (${\widehat{\beta }}_{group}$ = 2.929, p = 0.096, Hedges’ s g = 0.566). No other statistical differences were found between COVID-19 patients and healthy controls. Results of multivariate models per brain region of interest of the differences in fiber cross-section between groups are provided in Table 4.

**Table 4:** Results of multivariate models per brain region of interest of the differences in fiber cross-section between COVID-19 patients (n = 18) and healthy controls (n = 17).

Brain region	Estimate (SE)		Adjusted R-squared	Significance level	Effect size [95% CI] (Hedges’ s g)
Corpus callosum					
Motor segment	Intercept	0.077 (0.022)	0.050	0.001	0.552 [−0.133 to 1.237]
	Group	−0.051 (0.031)		0.104	
Parietal segment	Intercept	0.055 (0.027)	−0.020	0.047	0.188 [−0.486 to 0.862]
	Group	−0.021 (0.037)		0.573	
Premotor area & SMA	Intercept	0.082 (0.028)	−0.006	0.007	0.292 [−0.384 to 0.968]
	Group	−0.035 (0.039)		0.383	
Corticospinal tract*					
Left corticospinal tract	Intercept	NA	NA	NA	0.566 [−0.120 to 1.251]
	Group	2.929^+^ (0.028^++^)		0.096	
Right corticospinal tract	Intercept	NA	NA	NA	0.408 [−0.271 to 1.088]
	Group	1.526^+^ (0.013^++^)		0.225	
Medial lemniscus*					
Left medial lemniscus	Intercept	NA	NA	NA	0.571 [−0.115 to 1.257]
	Group	2.065^+^ (0.017^++^)		0.160	
Right medial lemniscus	Intercept	NA	NA	NA	0.293 [−0.382 to 0.969]
	Group	0.153^+^ (0.001^++^)		0.699	
Whole pyramidal tract*					
Left pyramidal tract	Intercept	NA	NA	NA	0.488 [−0.194 to 1.170]
	Group	2.181^+^ (0.020^++^)		0.149	
Right pyramidal tract	Intercept	NA	NA	NA	0.361 [−0.316 to 1.039]
	Group	1.196^+^ (0.010^++^)		0.282	

Group = COVID-19 patients, *SMA* supplementary motor area, *CI* confidence interval, *NA* not applicable, *SE* standard error.

*Univariate test used instead of multivariate test due to assumptions that were not fulfilled.

^+^F-value.

^++^Mean squared error.

**Figure 5:**
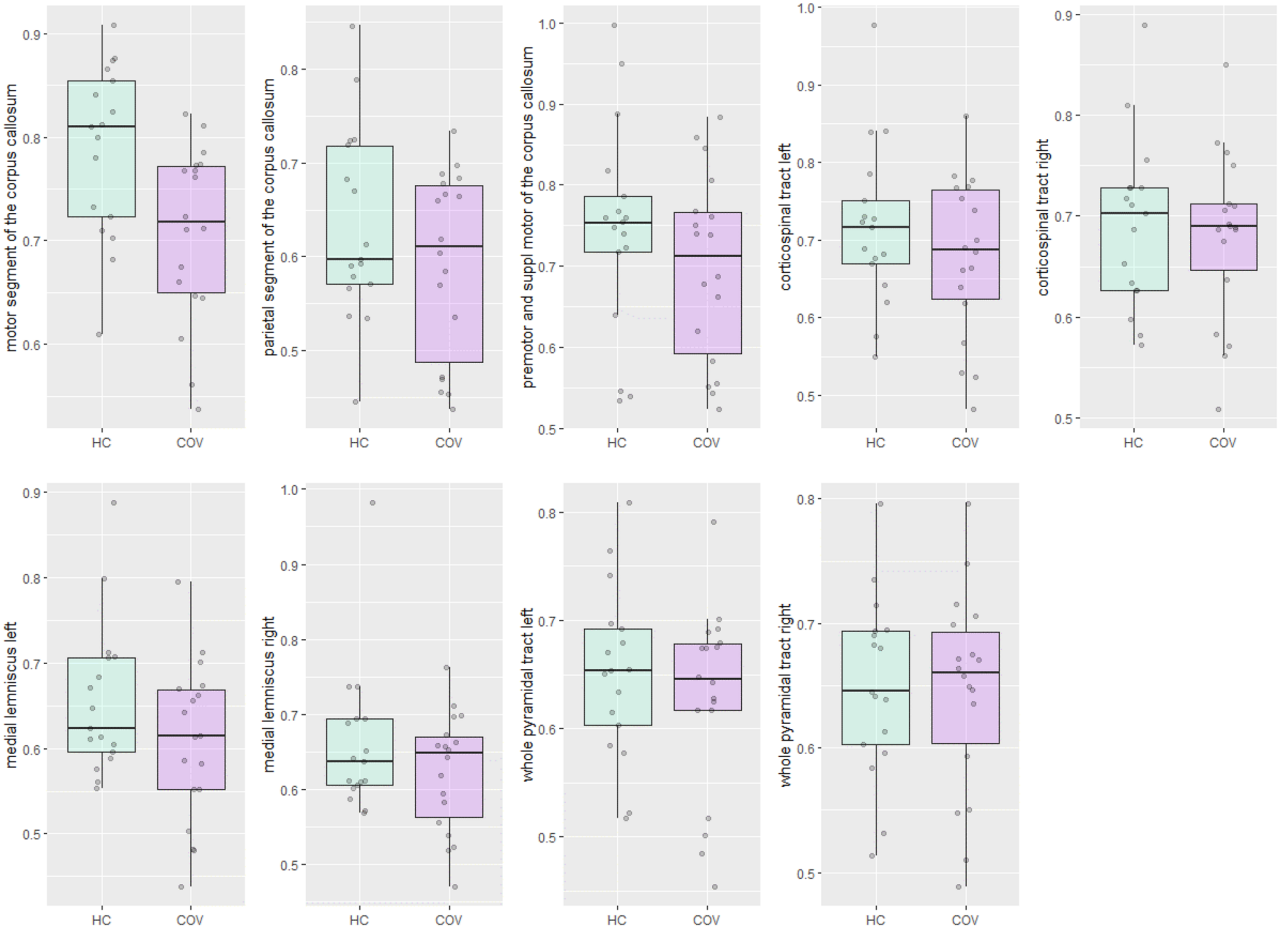
Fiber density cross-section of the motor segment of the corpus callosum, parietal segment of the corpus callosum, premotor and supplementary motor segment of the corpus callosum, bilateral medial lemnisci, bilateral whole pyramidal tracts and corticospinal tracts in COVID-19 patients (COV) and healthy controls (HC). Each facet presents the median and the IQR (box), Q1 − 1.5* IQR and Q3 + 1.5*IQR (whiskers), and individual observations (dots).

Figure 5 visualises FDC of the motor segment of the corpus callosum, parietal segment of the corpus callosum, premotor and supplementary motor segment of the corpus callosum, bilateral medial lemnisci, bilateral whole pyramidal tracts and corticospinal tracts in COVID-19 patients and healthy controls. A clear difference between COVID-19 patients and healthy controls was detected for the motor segment of the corpus callosum. This finding was confirmed by statistical testing, as can be seen in Table 5. Modelling showed a lower FDC in the motor segment of the corpus callosum in COVID-19 patients compared to healthy controls (${\widehat{\beta }}_{group}$ = −0.081, p = 0.007, Hedges’ s g = 0.945). No other statistical differences were found between COVID-19 patients and healthy controls.

**Table 5:** Results of multivariate models per brain region of interest of the differences in fiber density cross-section between COVID-19 patients (n = 18) and healthy controls (n = 17).

Brain region	Estimate (SE)		Adjusted R-squared	Significance level	Effect size [95% CI] (Hedges’ s g)
Corpus callosum					
Motor segment	Intercept	0.788 (0.020)	0.174	< 0.001	0.945 [0.236 to 1.653]
	Group	−0.081 (0.028)		**0.007**	
Parietal segment	Intercept	0.634 (0.024)	0.013	< 0.001	0.399 [−0.280 to 1.077]
	Group	−0.041 (0.034)		0.236	
Premotor area & SMA	Intercept	0.745 (0.030)	0.009	< 0.001	0.376 [−0.302 to 1.054]
	Group	−0.047 (0.042)		0.263	
Corticospinal tract*					
Left corticospinal tract	Intercept	NA	NA	NA	0.368 [−0.310 to 1.046]
	Group	1.239^+^ (0.013^++^)		0.274	
Right corticospinal tract	Intercept	NA	NA	NA	0.126 [−0.547 to 0.799]
	Group	0.144^+^ (0.001^++^)		0.706	
Medial lemniscus					
Left medial lemniscus	Intercept	0.701 (0.034)	−0.015	< 0.001	0.368 [−0.310 to 1.046]
	Group	−0.023 (0.032)		0.485	
Right medial lemniscus	Intercept	0.679 (0.019)	−0.031	< 0.001	0.126 [−0.547 to 0.799]
	Group	0.002 (0.026)		0.951	
Whole pyramidal tract*					
Left pyramidal tract	Intercept	NA	NA	NA	0.266 [−0.410 to 0.941]
	Group	0.646^+^ (0.004^++^)		0.427	
Right pyramidal tract	Intercept	NA	NA	NA	0.056 [−0.616 to 0.729]
	Group	0.029^+^ (< 0.001^++^)		0.865	

Group = COVID-19 patients, *SMA* supplementary motor area, *CI* confidence interval, *NA* not applicable, *SE* standard error.

*Univariate test used instead of multivariate test due to assumptions that were not fulfilled.

^+^F-value.

^++^Mean squared error.

Significant values are in bold.

### Aim 3: cognitive performance

**Figure 6:**
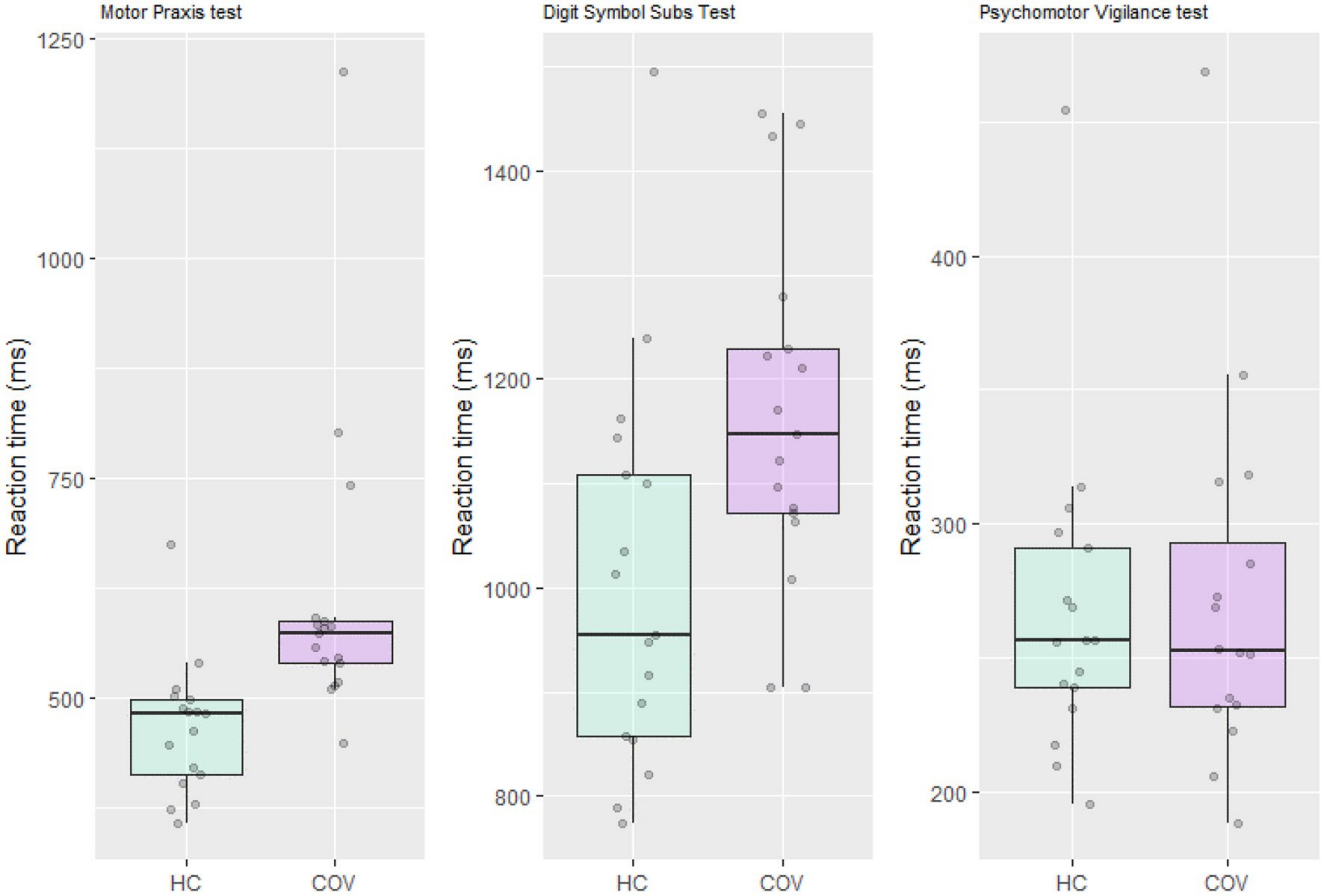
Boxplots of the cognitive performance expressed as reaction time on three cognitive tests in COVID-19 patients (purple) and healthy controls (green). Dots represent individual observations.

Figure 6 shows the boxplots of the cognitive performance, expressed as reaction time, on the motor praxis, digit symbol substitution and psychomotor vigilance tests in COVID-19 patients and healthy controls. This visualisation indicate a difference between groups on the Motor Praxis test and Digit Symbol substitution test.

Wilcoxon rank sum exact tests were subsequently used to statistically assess the difference in cognitive performance between COVID-19 patients and healthy controls. Median reaction times on the motor praxis test and digit symbol substitution test were significantly worse in COVID-19 patients compared to healthy controls (W = 28, p < 0.001, r = 0.688; W = 73, p = 0.013, r = 0.422, respectively). Despite the small sample size, medium to large effect sizes were found on the difference in cognitive performance for the motor praxis test and digit symbol substation test. Performance on the psychomotor vigilance test did not differ between COVID-19 patients and healthy controls (W = 136, p = 1, r = 0).

### Aim 4: associations between structural brain MRI measures and cognitive performance

Table 6 provides an overview of the two-group path model, grouping on patient type. This model looks at the associations between cognitive performance and the FD of the motor segment of the corpus callosum, parietal segment of the corpus callosum, premotor and supplementary motor segment of the corpus callosum, bilateral medial lemnisci, corticospinal tracts and whole pyramidal tracts. The outcome variables were the test score on the motor praxis test, digit symbol substitution test and psychomotor vigilance test, while the predictor variables comprised the aforementioned fiber bundles. The constructed model converged normally after 345 iterations and fits the data well according to the robust fit indices (${\chi }_{8}^{2}=1.029, p= 0.490;CFI=1.00, TLI=1.00, RMSEA= 0.000, SRMR=0.033)$.

**Table 6:** Results of the two-group path model on the associations between cognitive performance and fiber density for COVID-19 patients (n = 18) and healthy controls (n = 17).

		Healthy controls (estimate ± SE)	COVID-19 patients (estimate ± SE)	Difference (∆ estimate ± SE)	Significance level (p-value)
Regressions					
pvt ~	mot cc	1032.50 ± 186.17	395.92 ± 413.46	636.58 ± 453.44	0.160
	par cc	−323.87 ± 77.41	−548.10 ± 159.90	224.23 ± 177.65	0.207
	p&s cc	−550.50 ± 80.25	119.92 ± 264.96	−670.42 ± 276.85	**0.015**
	pyr L	1518.89 ± 649.21	−1580.07 ± 633.36	3098.96 ± 906.98	**0.001**
	pyr R	−719.65 ± 655.83	997.62 ± 539.19	−1717.27 ± 849.02	**0.043**
	med L	−765.05 ± 458.75	−566.86 ± 302.53	−198.20 ± 549.52	0.718
	med R	−297.98 ± 348.84	1892.19 ± 539.18	−2190.17 ± 642.18	**0.001**
dss ~	par cc	−1363.78 ± 453.38	−1135.90 ± 385.65	−227.88 ± 595.22	0.702
	pyr L	3657.56 ± 2157.73	−1375.86 ± 1197.76	5033.42 ± 2467.88	**0.041**
	pyr R	1888.98 ± 1793.90	1905.25 ± 700.83	−16.27 ± 1929.74	0.993
	med L	−3907.67 ± 1736.33	−1092.41 ± 777.36	−2815.26 ± 1902.40	0.139
	med R	1350.22 ± 1873.78	1959.50 ± 589.37	−609.27 ± 1964.28	0.756
mpt ~	mot cc	−307.89 ± 242.07	1914.10 ± 603.81	−2221.99 ± 650.53	**0.001**
	pyr L	186.81 ± 1030.89	−5583.54 ± 1655.62	5770.35 ± 1950.34	**0.003**
	pyr R	1475.33 ± 946.24	4322.29 ± 786.74	−2846.95 ± 1230.59	**0.021**
	med L	−1170.62 ± 736.09	−1588.70 ± 632.79	418.08 ± 970.70	0.667
	med R	73.69 ± 594.31	3799.03 ± 924.20	−3725.34 ± 1098.80	**0.001**
Covariances					
pvt ~~	dss	2474.50 ± 1088.38	−1550.362 ± 773.055		
	mpt	1761.63 ± 621.41	2126.565 ± 754.269		
dss ~~	mpt	4668.13 ± 1787.16	686.247 ± 1674.307		
Intercepts					
	pvt	270.11 ± 170.10	−271.81 ± 235.53		
	dss	125.81 ± 570.48	981.75 ± 363.27		
	mpt	351.39 ± 277.59	−1437.05 ± 350.88		
Variances					
	pvt	1301.23 ± 407.88	1723.39 ± 549.93		
	dss	11,890.18 ± 4147.64	9742.43 ± 3800.60		
	mpt	2813.03 ± 954.31	6105.12 ± 2076.29		

*mpt* motor praxis test, *dss* digit symbol substitution test, *pvt* psychomotor vigilance test, *mot cc* motor segment of the corpus callosum, *par cc* parietal segment of the corpus callosum, *p&s cc* premotor and supplementary motor segment of the corpus callosum, *med L* left medial lemniscus, *med R* right medial lemniscus, *pyr L* left whole pyramidal tract, *pyr R* right whole pyramidal tract, *SE* standard error.

Significant values are in bold.

New parameters were constructed. Here, a positive estimated difference denotes that the estimate of the COVID-19 patients was lower than that of the healthy controls. A negative estimated difference implies that the estimate of the COVID-19 patients score was higher than that of the healthy controls.

Comparing COVID-19 patients and healthy controls, we observed significantly different associations between the motor praxis test and the motor segment of the corpus callosum (${\widehat{\mathrm{mot cc}}}_{\Delta }$ = −2221.99, p = 0.001), bilateral pyramidal tracts (Left: ${\widehat{\mathrm{pyr L}}}_{\Delta }$ = 5770.35, p = 0.003, Right: ${\widehat{p\mathrm{yr R}}}_{\Delta }$ = −2846.95, p = 0.021) and right medial lemniscus (${\widehat{\mathrm{med R}}}_{\Delta }$ = −3725.34, p = 0.001). This was also the case for the associations between the digit symbol substitution test and the left pyramidal tract (${\widehat{\mathrm{pyr L}}}_{\Delta }$ = 5033.42, p = 0.041), and between the psychomotor vigilance test and the bilateral pyramidal tracts (Left: ${\widehat{\mathrm{pyr L}}}_{\Delta }$ = 3098.96, p = 0.001, Right: ${\widehat{\mathrm{pyr R}}}_{\Delta }$ = −1717.27, p = 0.043), the right medial lemniscus (${\widehat{\mathrm{med R}}}_{\Delta }$ = −2190.17, p = 0.001) and premotor and supplementary motor segment of the corpus callosum (${\widehat{\mathrm{p}\&\mathrm{s cc}}}_{\Delta }$ = −670.42, p = 0.015).

**Figure 7:**
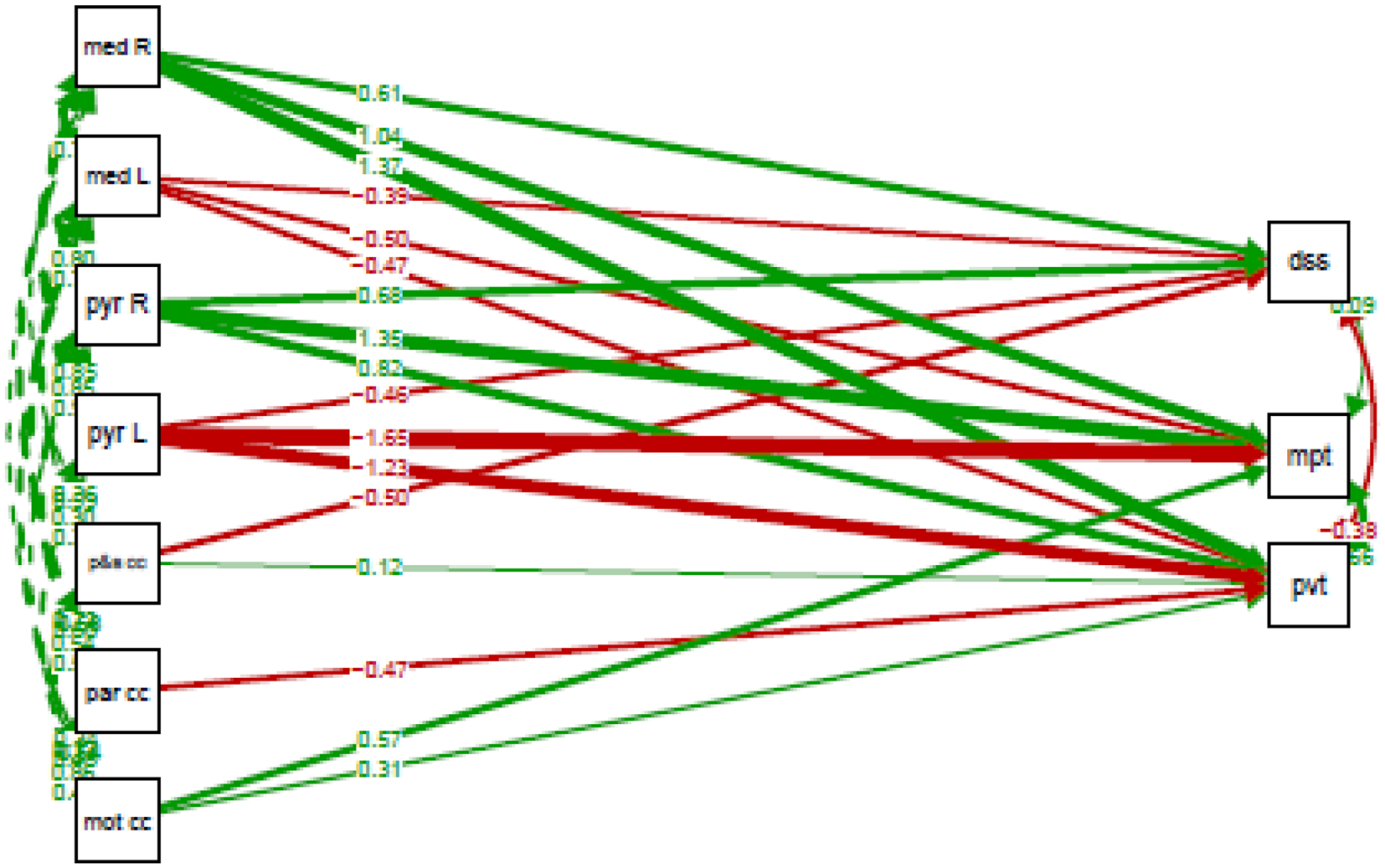
Associations between the cognitive performance on the motor praxis test (mpt), digit symbol substitution test (dss) and psychomotor vigilance test (pvt) and the fiber density of the motor segment of the corpus callosum (mot cc), parietal segment of the corpus callosum (par cc), premotor and supplementary motor segment of the corpus callosum (p&s cc), left and right medial lemnisci (med L & med R, respectively) and left and right whole pyramidal tracts (pyr L & pyr R, respectively) in COVID-19 patients. Positive estimates are indicated with green arrows, negative estimates with red arrows. The stronger the effect, the thicker the line of the arrows. Estimates are standardized. Single headed arrows are regressions, double headed arrows are covariances.

**Figure 8:**
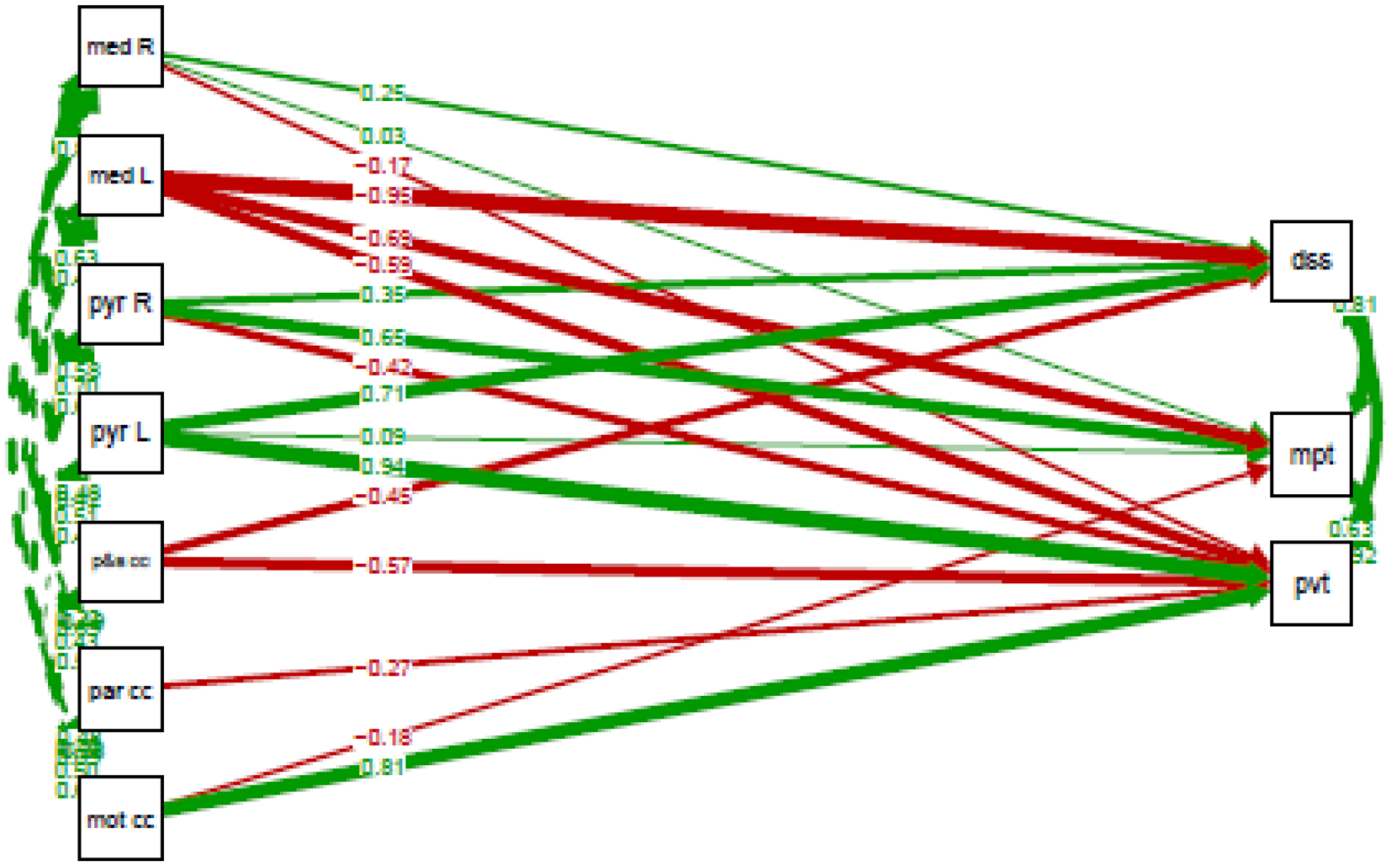
Associations in healthy controls between the cognitive performance on the motor praxis test (mpt), digit symbol substitution test (dss) and psychomotor vigilance test (pvt) and the fiber density of the motor segment of the corpus callosum (mot cc), parietal segment of the corpus callosum (par cc), premotor and supplementary motor segment of the corpus callosum (p&s cc), left and right medial lemnisci (med L & med R, respectively) and left and right whole pyramidal tracts (pyr L & pyr R, respectively). Positive estimates are indicated with green arrows, negative estimates with red arrows. The stronger the effect, the thicker the line of the arrows will be. Estimates are standardized. Single headed arrows are regressions, double headed arrows are covariances.

A visualisation of the associations between cognitive performance and FD is provided in Figs. 7 and 8, respectively for COVID-19 patients and controls.

## Discussion

This study explored the differences in structural whole-brain organisation, local white matter brain microstructure and cognitive performance between COVID-19 patients and healthy controls. Furthermore, we investigated whether differences in cognitive performance are associated with white-matter brain microstructure for COVID-19 patients and healthy controls. No differences in the structural whole-brain organisation were found between COVID-19 patients and healthy controls. Whole-brain FBA showed marginally significant differences in the corpus callosum and the subcortical white matter of the medial left precentral gyrus. Whole-brain FC showed marginally significant differences in the left cerebral peduncle and whole-brain FDC in the left pyramidal tract, the parietal segment of the corpus callosum, and the precentral gyrus of the subcortical white matter. On a tract-specific level, we found a reduction of FD and FDC in the motor segment of the corpus callosum and a marginal reduction in FC in the left corticospinal tract among COVID-19 patients indicating differences in intra-axonal volume (e.g. axonal loss) and macroscopic cross-sectional axonal size compared to healthy controls, respectively^31^. The motor segment of the corpus callosum is the main interhemispheric connection between the primary motor cortices, whereas the corticospinal tract transmits motor-related impulses from the cerebral cortex to the spinal tract^40^. Consequently, the differences in FD, FC and FDC could indicate a changed ability to relay information impacting cognitive performance^40^. Regarding cognitive performance, we found that performance on the motor praxis and digit symbol substitution tests was worse in COVID-19 patients than the healthy controls, indicating reduced sensory-motor speed and problems with complex tracking and visual scanning^41^.

The associations between the microstructural brain structure (i.e. fiber density) and performance on the cognitive tasks were examined using structural equation modelling. Significant differences in associations were found between the performance on the motor praxis test, and the motor segment of the corpus callosum, the left and right whole-pyramidal tracts and the right medial lemniscus in COVID-19 patients compared to the group of healthy controls (Table 6). The associations between the digit symbol substitution test and the left whole-pyramidal tract, and between the psychomotor vigilance test and parietal segment of the corpus callosum, the right medial lemniscus and the left and right whole pyramidal tracts also significantly differed between COVID-19 patients and the healthy controls (Table 6). These results suggest that COVID-19 may induce structural white-matter brain changes that are likely to induce cognitive problems.

Bispo et al. found a reduction of fibre density in recovered COVID-19 patients compared to healthy controls in the posterior genu and rostral body of the corpus callosum, the arcuate fasciculus, the cingulum, the fornix, the inferior frontal-occipital fasciculus, the inferior and superior longitudinal fasciculus, the uncinate fasciculus, the corona radiata, and corticospinal tracts^33^. These reductions in fibre density were also correlated with a worse outcome for COVID-19 patients on reaction time and visual memory tests^33^. These results are consistent with our results. However, caution is needed when comparing our results to those of Bispo et al. Their results might be less specific to the white matter microstructure because their acquisition protocol constituted only 32 directions with a b-value of 800 s/mm^2^^33^. This is below the advised b-value > 2000s/mm2 for CSD, resulting in lower angular resolution and less specificity for restricted diffusion within the axons^55^. The population difference between the two studies must also be pointed out. Bispo et al. (2022) mainly included non-hospitalised individuals tested three months after their COVID-19 recovery, while the present study included hospitalised patients tested one month after recovery^33^. This prompts the question about the influence of hospitalisation length and related disease severity on the effect of COVID-19 on brain structure and cognitive performance^56^. Consequently, more research is needed to determine whether experiencing a COVID-19 infection affects the brain structure and cognitive performance while accounting for hospitalisation-related factors such as the degree of hospitalisation (e.g. residing on an intensive care unit, use of artificial breathing machines) and hospitalisation length.

A strength of this study is that both groups were homogeneous regarding vaccination status, as no vaccination was possible at the time of the data collection, thus limiting the selection and information bias. Hence, vaccination status has no confounding effect^57–63^. Future COVID-19 studies should account for the influence of vaccination status as a possible confounding factor. A study limitation is our small sample size. Therefore, we attached more significance to the visualisations (i.e. data distributions) and effect sizes than to the p-values. In our analyses, we also neutralised this limitation using statistical tests and models designed to control for a small sample size. Additionally, the small sample size limits performing a covariates analysis to correct for possible baseline differences, despite the study groups being matched by age. Future research should implement such analysis to acquire a more comprehensive understanding of the groups. Furthermore, caution is needed when interpreting the results of the white-matter microstructure. Due to the limited sample size, we could not control for brain-size scaling effects. Therefore, we decided not to include the FC and FDC in our last hypothesis (i.e. associations), because of to their intrinsic relation to intracranial volume^30,31^. Inclusion of these measures in future studies might shed a broader light on the effects of COVID-19 on white-matter macro-structure. A last limitation comprises our cross-sectional study design. This design limits us from examining the differences between COVID-19 patients and healthy controls at one given point in time. Future research should include the longitudinal follow-up of brain structure and cognitive performance in COVID-19 patients compared to healthy controls to unravel interactions between brain structure and cognitive performance over time^64^.

## Conclusion

This study found no differences in whole-brain organisation in recovered hospitalised COVID-19 patients compared to healthy controls but did find a difference in fiber density and fiber density cross-section of the motor segment of the corpus callosum. Recovered hospitalised COVID-19 patients also showed a lower cognitive performance in terms of sensory-motor speed, complex tracking and visual scanning. Cognitive performance and white-matter microstructure appeared to be associated and significantly differed between COVID-19 patients and healthy controls. Future research is needed to further investigate this association and its underlying mechanisms and investigate how cognitive performance and microstructural brain changes evaluate over time.

## Data Availability

The data that support the findings of this study are available on request from the corresponding author. The data are not publicly available due to privacy or ethical restrictions.
