# Author Correction: A cross-sectional case–control study on the structural connectome in recovered hospitalized COVID-19 patients

**DOI:** 10.1038/s41598-023-44726-y

**Published:** 2023-11-03

**Authors:** Elke Lathouwers, Ahmed Radwan, Jeroen Blommaert, Lara Stas, Bruno Tassignon, Sabine D. Allard, Filip De Ridder, Elisabeth De Waele, Nicole Hoornaert, Patrick Lacor, Rembert Mertens, Maarten Naeyaert, Hubert Raeymaekers, Lucie Seyler, Anne-Marie Vanbinst, Lien Van Liedekerke, Jeroen Van Schependom, Peter Van Schuerbeek, Steven Provyn, Bart Roelands, Marie Vandekerckhove, Romain Meeusen, Stefan Sunaert, Guy Nagels, Johan De Mey, Kevin De Pauw

**Affiliations:** 1 Human Physiology and Sports Physiotherapy Research Group, Vrije Universiteit Brussel, Brussels, Belgium; 2 Department of Imaging and Pathology, Translational MRI , KU Leuven, Leuven, Belgium; 3 Department of Oncology, KU Leuven, Leuven, Belgium; 4 Core Facility‑Support for Quantitative and Qualitative Research (SQUARE), Vrije Universiteit Brussel, Brussels, Belgium; 5 Biostatistics and Medical Informatics Research Group, Faculty of Medicine and Pharmacy, Department of Public Health, Vrije Universiteit Brussel, Brussels, Belgium; 6 Infectious Diseases Unit, Department of Internal Medicine, UZ Brussel, Jette, Belgium; 7 Department of Radiology and Magnetic Resonance, UZ Brussel, Brussels, Belgium; 8 Intensive Care Unit, UZ Brussel, Jette, Belgium; 9 Department of Electronics and Informatics (ETRO), Vrije Universiteit Brussel, Brussels, Belgium; 10 Artifcial Intelligence and Modelling in Clinical Science, Vrije Universiteit Brussel, Brussels, Belgium; 11 Department of Anatomical Research and Clinical Studies (ARCS), Vrije Universiteit Brussel, Brussels, Belgium; 12 Faculty of Psychology and Educational Sciences, Vrije Universiteit Brussel, Brussels, Belgium; 13 Faculty of Medicine and Pharmaceutical Sciences, Vrije Universiteit Brussel, Brussels, Belgium; 14 Faculty of Arts and Philosophy, University of Ghent, Ghent, Belgium; 15 BruBotics, Vrije Universiteit Brussel, Brussels, Belgium; 16 Strategic Research Program ‘Exercise and the Brain in Health & Disease: The Added Value of Human‑Centered Robotics’, Vrije Universiteit Brussel, Brussels, Belgium; 17 Department of Radiology, UZ Leuven, Leuven, Belgium

Correction to: *Scientific Reports* 10.1038/s41598-023-42429-y, published online 21 September 2023

The original version of this Article contained an error, where the given names were not spelled out for the authors Filip De Ridder, Elisabeth De Waele, Nicole Hoornaert, Patrick Lacor, Rembert Mertens, Anne-Marie Vanbinst, Romain Meeusen, Guy Nagels, and Johan De Mey.

The original Article has been corrected.

